# Superiority of Formalin-Fixed Paraffin-Embedded Brain Tissue for *in vitro* Assessment of Progressive Supranuclear Palsy Tau Pathology With [^*18*^F]PI-2620

**DOI:** 10.3389/fneur.2021.684523

**Published:** 2021-07-02

**Authors:** Marie Willroider, Sigrun Roeber, Anja K. E. Horn, Thomas Arzberger, Maximilian Scheifele, Gesine Respondek, Osama Sabri, Henryk Barthel, Marianne Patt, Olena Mishchenko, Andreas Schildan, André Mueller, Norman Koglin, Andrew Stephens, Johannes Levin, Günter U. Höglinger, Peter Bartenstein, Jochen Herms, Matthias Brendel, Leonie Beyer

**Affiliations:** ^1^Department of Nuclear Medicine, University Hospital of Munich, LMU Munich, Munich, Germany; ^2^Center for Neuropathology and Prion Research, LMU Munich, Munich, Germany; ^3^Institute of Anatomy and Cell Biology, LMU Munich, Munich, Germany; ^4^German Center for Neurodegenerative Diseases (DZNE), Munich, Germany; ^5^Department of Neurology, Hannover Medical School, Hanover, Germany; ^6^Department of Nuclear Medicine, University Hospital Leipzig, Leipzig, Germany; ^7^Life Molecular Imaging GmbH, Berlin, Germany; ^8^Department of Neurology, University Hospital Munich, LMU Munich, Munich, Germany; ^9^Munich Cluster for Systems Neurology (SyNergy), Munich, Germany; ^10^Department of Neurology, Technical University Munich, Munich, Germany

**Keywords:** tau, autoradiography, immunohistochemistry, progressive supranuclear palsy, PI-2620

## Abstract

**Objectives:** Autoradiography on brain tissue is used to validate binding targets of newly discovered radiotracers. The purpose of this study was to correlate quantification of autoradiography signal using the novel next-generation tau positron emission tomography (PET) radiotracer [^18^F]PI-2620 with immunohistochemically determined tau-protein load in both formalin-fixed paraffin-embedded (FFPE) and frozen tissue samples of patients with Alzheimer's disease (AD) and Progressive Supranuclear Palsy (PSP).

**Methods:** We applied [^18^F]PI-2620 autoradiography to postmortem cortical brain samples of six patients with AD, five patients with PSP and five healthy controls, respectively. Binding intensity was compared between both tissue types and different disease entities. Autoradiography signal quantification (CWMR = cortex to white matter ratio) was correlated with the immunohistochemically assessed tau load (AT8-staining, %-area) for FFPE and frozen tissue samples in the different disease entities.

**Results:** In AD tissue, relative cortical tracer binding was higher in frozen samples when compared to FFPE samples (CWMR_frozen_ vs. CWMR_FFPE_: 2.5-fold, *p* < 0.001), whereas the opposite was observed in PSP tissue (CWMR_frozen_ vs. CWMR_FFPE_: 0.8-fold, *p* = 0.004). In FFPE samples, [^18^F]PI-2620 autoradiography tracer binding and immunohistochemical tau load correlated significantly for both PSP (*R* = 0.641, *p* < 0.001) and AD tissue (*R* = 0.435, *p* = 0.016), indicating a high agreement of relative tracer binding with underlying pathology. In frozen tissue, the correlation between autoradiography and immunohistochemistry was only present in AD (*R* = 0.417, *p* = 0.014) but not in PSP tissue (*R* = −0.115, *p* = n.s.).

**Conclusion:** Our head-to-head comparison indicates that FFPE samples show superiority over frozen samples for autoradiography assessment of PSP tau pathology by [^18^F]PI-2620. The [^18^F]PI-2620 autoradiography signal in FFPE samples reflects AT8 positive tau in samples of both PSP and AD patients.

## Introduction

Many neurodegenerative diseases are still lacking options to reliably diagnose the causal neuropathology *in vivo*. Facing the huge and growing number of patients suffering from those diseases, it will be important for global health care systems to improve diagnosis and to stratify individuals at risk in order to provide the best patient management and offer possible inclusion to therapy studies. In addition to characterization of the clinical phenotype, supportive *in vivo* biomarkers have been introduced to many diagnostic schemes to describe the neuropathological correlates of the diseases (1).

Tauopathies form the major group of adult-onset neurodegenerative diseases including Alzheimer's disease (AD) and frontotemporal lobar degeneration (FTLD-tau) spectrum diseases including some atypical Parkinsonian syndromes. *In vivo* visualization of tau deposits is now facilitated by positron emission tomography (PET) using different tau-targeting tracers (2–4), but reliable quantification of intracerebral tau burden remains difficult. First-generation PET tau tracers suffered from large inter- and intra-case variability due to off-target binding (5–7). Therefore, it is crucial to carefully validate novel next-generation tau tracers *in vitro* on human postmortem brain tissue of neuropathologically confirmed tauopathies. While the mixed three-/four-repeat (3R/4R) tau pathology of AD has been shown to be detectable with various tau-specific radiotracers (8–11), detection of 4R tau in progressive supranuclear palsy (PSP) is more challenging and only few examples of detectable binding have been presented so far (12, 13). In a subsample of those cases, blocking with excessive nonlabeled [^19^F]PI-2620 indicated specific binding in PSP (12).

Although many neuropathology departments aim to preserve both frozen and formalin-fixed paraffin-embedded (FFPE) samples, the preservation of brain samples is not standardized due to different resources and available procedures. The formalin-fixation is broadly available, does not require expensive equipment and the storage is convenient. Furthermore, it is tissue-conserving and does not interact with the primary structure of cells, but long storage of tissue in formalin leads to denaturation of proteins and deoxyribonucleic acids (14). Many studies suggest freezing as a better way to preserve molecular targets for analyses requiring very high resolution, such as examination of protein structures (15). However, the applied freezing procedure including the reachable freezing time has a great impact on the quality of frozen tissue samples and can diminish the integrity of proteins. Also, different antibodies have different binding characteristics in FFPE/ frozen samples (16). Taken together, the superiority of freezing over formalin fixation could not be confirmed in a head-to-head comparison using various immunohistochemistry antibodies (16).

In this regard, autoradiography (ARG) aiming to detect aggregated tau has been successfully conducted on paraffin-embedded (17) and frozen brain tissue (9, 11). ARG with β-amyloid radiotracers was also successfully performed with both types of tissue samples (18–21). However, head-to-head comparisons of ARG in FFPE and frozen tissue are rare. A small subsample of six patients with AD showed that despite a good correlation between both techniques, the binding [pmol/mg] of the radioligand ([^3^H]PiB) to the FFPE samples was only 43 ± 24% of that observed in the corresponding frozen tissue samples (19). In a study investigating the β-amyloid PET tracer [^18^F]florbetapir, frozen and FFPE brain sections were analyzed head-to-head, both showing strong quantitative correlations in the gray matter between radiotracer binding (optical density of the signal) and β-amyloid (% area) to immunohistochemistry (21). Supportive *in vitro* ARG data of our recent [^18^F]PI-2620 investigation in PSP showed a detectable and blockable tracer signal in FFPE sections of the basal ganglia and the frontal cortex (12). However, other groups were not able to see elevated [^18^F]PI-2620 binding in frozen PSP tissue (10, 11).

Therefore, the objective of this study was to systematically investigate the impact of tissue sample preparation on radiotracer binding in [^18^F]PI-2620 ARG of PSP and AD samples. We aimed to compare ARG quantification of both tauopathies and healthy controls (HC) between FFPE and frozen tissue. Furthermore, ARG quantification of [^18^F]PI-2620 binding to both tissue types was correlated with the immunohistochemically assessed tau load.

## Materials and Methods

### Brain Tissue Samples

Tissue samples of all autopsy cases investigated were provided by the Neurobiobank Munich, Ludwig-Maximilians-University (LMU) Munich. They were collected according to the guidelines of the local ethical committee and usage of the material for this project was additionally approved (application number 19-244). Autopsies and subsequent analysis of brain pathology were performed according to standardized protocols (22–24). During autopsy, one hemisphere of the brain is fixed in formalin and the other hemisphere is sliced and frozen at −80°C. Samples of frozen and formalin-fixed tissue of the frontal cortex (Gyrus frontalis medius) from six patients with AD, five patients with PSP and five HC were included in the analysis. Only cases without relevant co-pathology in the cortical target region (negative for α-synuclein and Aβ in PSP/ HC) were selected from the database. Cortical tissue was chosen to evaluate binding in a brain area with limited off-target sources and to be able to directly compare AD and PSP tissue. Furthermore, collection of frozen basal ganglia material can be accompanied by destruction of sample material in the surrounding brain areas. Therefore, we compared both techniques in frontal cortex material which is affected in both diseases and is relatively easy to obtain in both FFPE and frozen material (stored at −80°C) from the same patients by collecting the respective samples from both hemispheres.

### Immunohistochemistry and Tau Load Quantification

**Figure 1:**
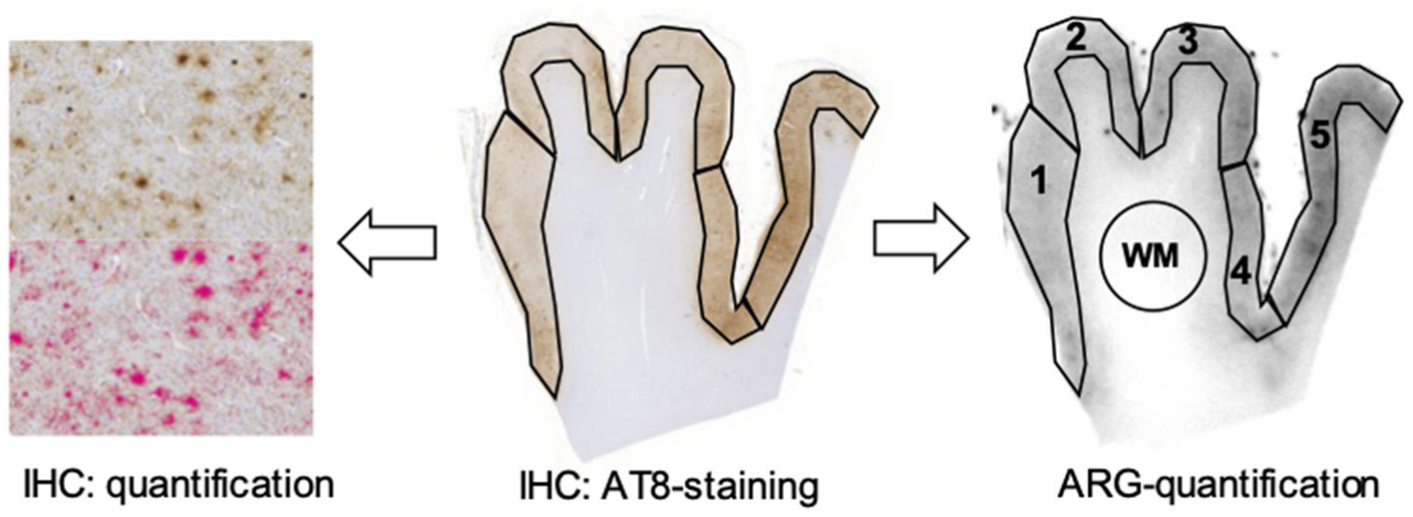
Tau load quantification in immunohistochemistry (IHC) samples with AT8-staining based on color thresholds on the left (upper image: original AT8 image, bottom image: all parts within the threshold marked in pink) and quantification of autoradiography signal (ARG) in corresponding frontal cortex brain areas (on the right). The subcortical white matter (WM) was used as a reference region.

Immunhistochemistry was performed on 4 μm thick paraffin sections of formalin-fixed tissue and on 10 μm thick brain sections of frozen brain tissue using standard techniques. The immunohistochemical tau-staining was performed semi-automatically on a BenchMark device (Ventana, now Hoffmann-LaRoche, Basel, Switzerland) with mouse monoclonal AT8 antibody raised against hyperphosphorylated tau (Ser202/Thr205, 1:200, Invitrogen/Thermofisher, Carlsbad, CA, USA) on adjacent sections of those used in the ARG. The immunostained sections were digitized at 20x magnification with a Mirax Midi scanner (Zeiss, Carl Zeiss MicroImaging GmbH, Jena, Germany). Five cortical gray matter regions of interest were drawn manually and the tau load (in %) was quantified using the Pannoramic Viewer (1.15.2) software with HistoQuant (3DHISTECH, Budapest, Hungary) based on color threshold values (see Figure 1). All brain sections included are shown in the Supplementary Material.

### *In vitro* ARG

[^18^F]PI-2620 was synthesized as previously described (25). For each subject and sample type (FFPE, frozen), ≥6 sections (consecutive to immunohistochemistry) were prepared for ARG and ≥4 artifact-free sections (no freezing artifacts, no artificial tracer retention due to insufficient washing, intact brain tissue) were used for analysis (see Supplementary Material for all used and excluded brain sections). The sections (both FFPE and frozen) were incubated with [^18^F]PI-2620 (21.6 μCi/ml after dilution to a volume of 50 ml with phosphate buffered saline solution, pH 7.4, specific activity 480±90 GBq/μmol) for 45 min. Washing was performed by 30% ethanol/PBS for 1 min, 70% ethanol/PBS for 2 min and PBS for 1 min. After drying at room temperature for 60 min, the sections were placed on Fujifilm BAS cassette2 2025 imaging plates. The plates were exposed for 12 h and then scanned at 25.0 μm resolution with the Elysia-raytest equipment (CR-35 BIO, Dürr Medical, Bietigheim-Bissingen, Germany). Resulting images were analyzed with a dedicated software (AIDA image analysis, V4.50, Elysia-raytest, Straubenhardt, Germany). Five cortical gray matter regions of interest were drawn manually on each sample with AT8 staining of adjacent sections serving for precise anatomical definition (but blinded to quantification of AT8). AT8 negative white matter (assessed visually) served as reference region (circle area) and for calculation of tracer uptake ratios between target and reference regions. The background of the photo plate was subtracted before quantification. Analysis resulted in ARG binding ratios between frontal cortex and white matter (CWMR) for both FFPE (CWMR_FFPE_) and frozen samples (CWMR_frozen_). For illustration of the ARG signal quantification see Figure 1. All brain sections included are shown in the Supplementary Material.

### Statistical Analysis

GraphPad Prism (version 8.4.3, GraphPad Software Inc., San Diego, CA, USA) was used for statistical analysis and illustration of results. Demographics of patient groups (age, postmortem delay, fixation time) were compared using a Kruskal-Wallis test for multiple comparisons). Immunohistochemical tau load and ARG binding ratios were compared between AD, PSP, and HC for both tissue types by a Welch analysis of variance (after testing for homogeneity of variances by Brown-Forsythe) and Dunnett's T3 multiple comparisons test. ARG signal quantification of FFPE and frozen samples were correlated with corresponding immunohistochemical tau load in the same tissue type samples by a linear regression with error bars of ARG ratio quantification results (separately for AD and PSP). A significance level of *p* < 0.05 was applied in all analyses.

## Results

### Demographics

Table 1 summarizes demographic, clinical, and neuropathological characteristics of patients included. The cohort consisted of six subjects with AD (mean age 75 years, range 63–88, 4 female), five subjects with PSP (mean age 76 years, range 67–85, 4 female) and five HC (mean age 57, range 46–85, 2 female). The mean age was not significantly different between groups (AD: 75 ± 8 y, PSP: 76 ± 9 y, HC: 58 ± 16 y). The postmortem delay was equal in all groups (AD: 27.8 ± 15.0 h, PSP: 18.4 ± 12.9 h, HC: 20.6 ± 2.2 h). When compared to HC, the fixation times were longer in AD (*p* = 0.025) and PSP patients (*p* = 0.066), but not between AD and PSP (AD: 135 ± 77 d, PSP: 117 ± 68 d, HC: 8 ± 4 d).

**Table 1:** Patient characteristics.

**Autopsy diagnosis category**	**ID**	**Clinical diagnosis**	**Cause of death**	**Age at death (y)**	**Gender**	**Autopsy diagnosis**	**ABC score**	**Frontal cortex: Aβ/α-syn/TDP-43/FUS**	**Postmortem delay (h)**	**Fixation time (d)**	**APOE**
AD	1	AD	Pneumonia	75	Female	AD, CAA	A3/B3/C3	+ /–/–/ n.s.	<12	138	E3/E3
	2	AD	n.s.	79	Female	AD, CAA, vascular lesions	A3/B3/C3	+ /–/–/ n.s.	20	44	E3/E4
	3	CBS	n.s.	73	Female	AD, CAA	A3/B3/C3	+/–/–/ n.s.	36	>60	E4/E4
	4	Dementia n.s	Pyelonephritis	88	Female	AD, vascular lesions	A3/B3/C3	+ /–/–/ n.s.	54	229	E3/E4
	5	AD, PD	n.s.	63	Male	AD, CAA, vascular lesions	A3/B3/C3	+ /–/–/ n.s.	21	76	E3/E3
	6	AD	n.s.	70	Male	AD, CAA	A3/B3/C3	+ /–/–/ –	24	190	E3/E3
PSP	7	PSP	n.s.	85	Female	PSP	A0/B1/C0	–/–/ n.s. / n.s.	15	n.s.	E2/E3
	8	AD+PD	Terminal dementia	84	Female	PSP, atherosclerosis	A0/B0/C0	–/–/ n.s. / n.s.	8	n.s.	n.s.
	9	FTD (CBS)	Cerebral hemorrhage	78	Female	PSP, AGD, multiple cerebral hemorrhages	A0/B2/C0	– /–/–/ n.s.	24	76	E3/E3
	10	PSP	Terminal dementia	67	Female	PSP	A0/B0/C0	–/–/–/ –	7	80	E3/E3
	11	PSP	Cachexia	67	Male	PSP	A0/B1/C0	–/–/–/ –	38	195	n.s.
HC	12	NNPD,	Pulmonary embolism	46	Female	NNPF	A0/B1/C0	–/–/ n.s. / n.s.	17	3	E2/E3
	13	NNPD	Sudden cardiac death	53	Male	NNPF	n.s./B1/C0	–/–/ n.s. / n.s.	22	5	E3/E3
	14	NNPD, CHD, DM	MI	61	Male	NNPF	A0/B1/C0	–/–/–/ –	22	7	E3/E3
	15	NNPD, CHD	MI	85	Female	NNPF	A0/B1/C0	–/–/ n.s. / n.s.	20	13	E3/E3
	16	NNPD, CHD	MI	46	Male	NNPF	n.s./B0/C0	–/–/ n.s. / n.s.	22	10	E3/E4

*AD, Alzheimer's disease; PSP, progressive supranuclear palsy; HC, healthy control; CBS, corticobasal syndrome; PD, Parkinson's disease; FTD, frontotemporal dementia; NNPD, no neurological/psychiatric disease; CHD, coronary heart disease; DM, diabetes mellitus; MI, myocardial infarction; CAA, cerebral amyloid angiopathy; AGD, agyrophilic grain disease; NNPF, no neuropathological findings; ABC-score, Amyloid Braak CERAD (Consortium to Establish a Registry for Alzheimer's Disease); Aβ, amyloid-β; α-syn, α-synuclein; TDP-43, Transactive response DNA binding protein 43 kDa; FUS, fused in sarcoma; n.s., not specified*.

All cases were lacking relevant copathology in the cortical target region (negative for α-synuclein and Aβ for PSP/HC). All AD patients had high Alzheimer's disease neuropathologic change (ADNC) levels (A3, B3, C3) (26).

### Visual Assessment of *in vitro* [^18^F]PI-2620 Binding and Immunohistochemical AT8 Staining

**Figure 2:**
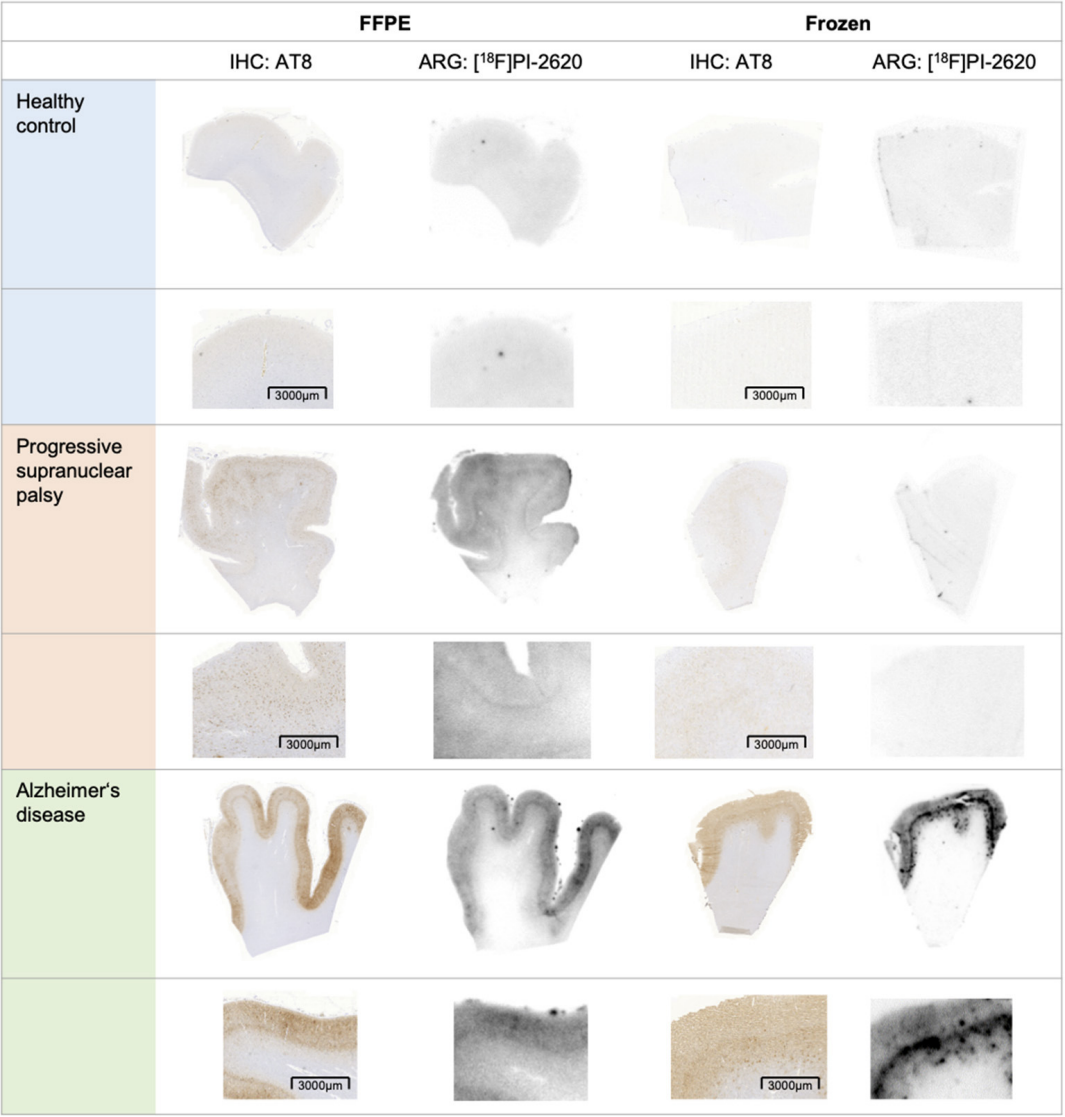
Exemplary tau-immunohistochemistry (left corresponding sections) and autoradiography (right corresponding sections) of frontal cortex sections of both tissue types (FFPE on the left, frozen on the right) in a healthy control (both no. 15), progressive supranuclear palsy (both no. 8) and Alzheimer's disease (FFPE: no. 4, frozen: no. 3). Upper row: section overview, lower row: zoom. FFPE, formalin-fixed paraffin-embedded; IHC, immunohistochemistry; ARG, autoradiography.

Visual comparison between *in vitro* [^18^F]PI-2620 binding and AT8 staining revealed high concordance for PSP and AD cases in FFPE samples. *In vitro* [^18^F]PI-2620 binding in the cortex of PSP tissue was consistently lower when compared to AD tissue. Exemplary FFPE and frozen samples of one HC, PSP, and AD patient each are shown in Figure 2.

### Comparison Between FFPE and Frozen-Tissue Samples

**Figure 3:**
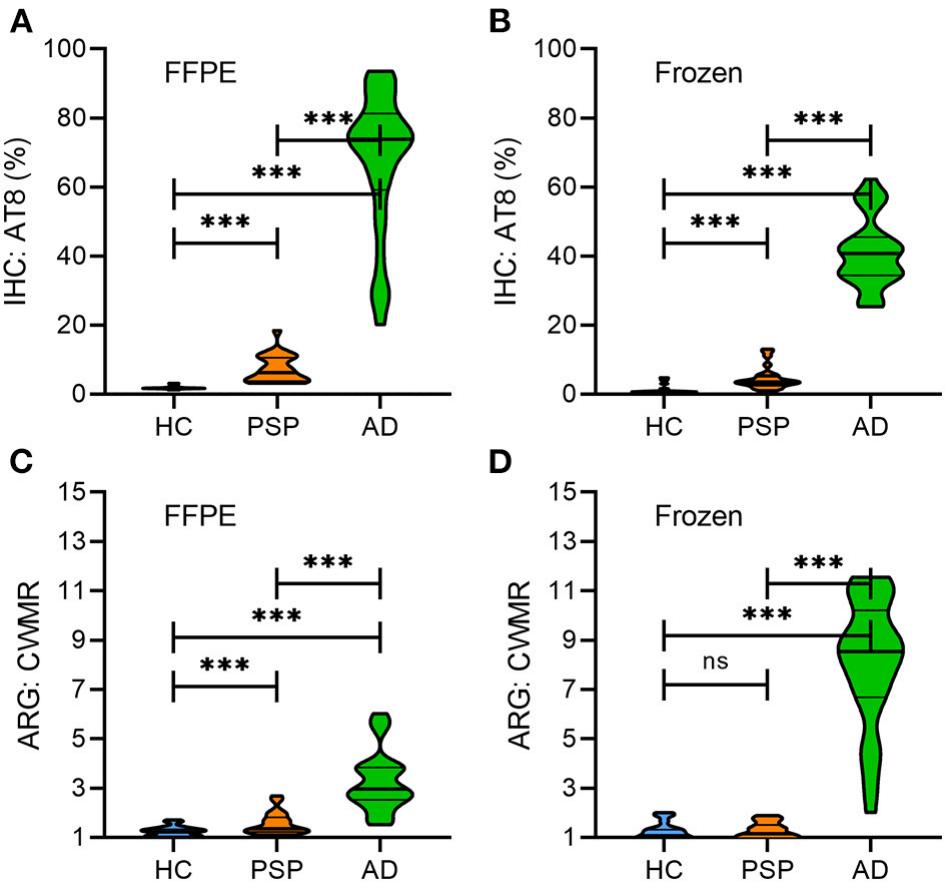
Quantitative comparison of immunohistochemistry (% tau load) and autoradiography (cortex to white matter ratio) between HC, PSP and AD in FFPE **(A,C)** and frozen **(B,D)** tissue samples. Violin plots represent the distribution of data with the median and quartiles. HC, healthy controls, PSP, progressive supranuclear palsy; AD, Alzheimer's disease; FFPE, formalin-fixed paraffin-embedded; IHC, immunohistochemistry; ARG, autoradiography; CWMR, cortex to white matter ratio; ***p < 0.001; n.s., not significant.

Table 2 provides an overview of immunohistochemistry and ARG results. The immunohistochemically determined tau load (AT8 staining, separately for FFPE and frozen tissue) was significantly higher in AD tissue when compared to PSP (AT8_FFPE_: 9.7-fold, *p* < 0.001; AT8_frozen_: 7.4-fold, *p* < 0.001) as illustrated in Figures 3A,B. For both FFPE and frozen-tissue samples and in accordance with the immunohistochemical tau load, cortical [^18^F]PI-2620 binding ratios were significantly higher in AD tissue when compared to PSP (CWMR_FFPE_: 2.1-fold, *p* < 0.001; CWMR_frozen_: 6.8-fold, *p* < 0.001) and HC (CWMR_FFPE_: 2.8-fold, *p* < 0.001; CWMR_frozen_: 6.8-fold, *p* < 0.001).

**Table 2:** Immunohistochemistry and autoradiography results.

	**Healthy controls**	**Progressive supranuclear palsy**	**Alzheimer's disease**
AT8_FFPE_ (% ± SD)	1.8 ± 0.4	7.2 ± 0.4	67.5 ± 20.3
AT8_Frozen_ (% ± SD)	1.2 ± 1.3	4.6 ± 3.3	41.1 ± 10.0
CWMR_FFPE_ (± SD)	1.2 ± 0.2	1.6 ± 0.4	3.3 ± 1.2
CWMR_Frozen_ (± SD)	1.2 ± 0.4	1.2 ± 0.3	8.1 ± 2.6

*Presented data are percentages of tau load for immunohistochemistry and cortex to white matter ratios (CWMR) for autoradiography results. FFPE, formalin-fixed paraffin-embedded; SD, standard deviation*.

In PSP, significantly higher cortical [^18^F]PI-2620 binding ratios compared to HC were only evident in paraffin-embedded samples (CWMR_FFPE_: 1.3-fold, *p* < 0.001) but not in frozen-tissue samples (CWMR_frozen_: 1.0-fold, *p* = n.s.) (Figures 3C,D).

Comparing FFPE and frozen samples, relative [^18^F]PI-2620 binding was higher in frozen AD samples when compared to FFPE (CWMR_frozen_ vs. CWMR_FFPE_: 2.5-fold, *p* < 0.001), whereas in frozen PSP tissue the relative binding was lower when compared to FFPE tissue (CWMR_frozen_ vs. CWMR_FFPE_: 0.8-fold, *p* = 0.004). All ARG binding ratio differences between groups are illustrated in Figures 3C,D.

### Quantitative Correlation of *in vitro* [^18^F]PI-2620 Binding and Immunohistochemical Tau Load

**Figure 4:**
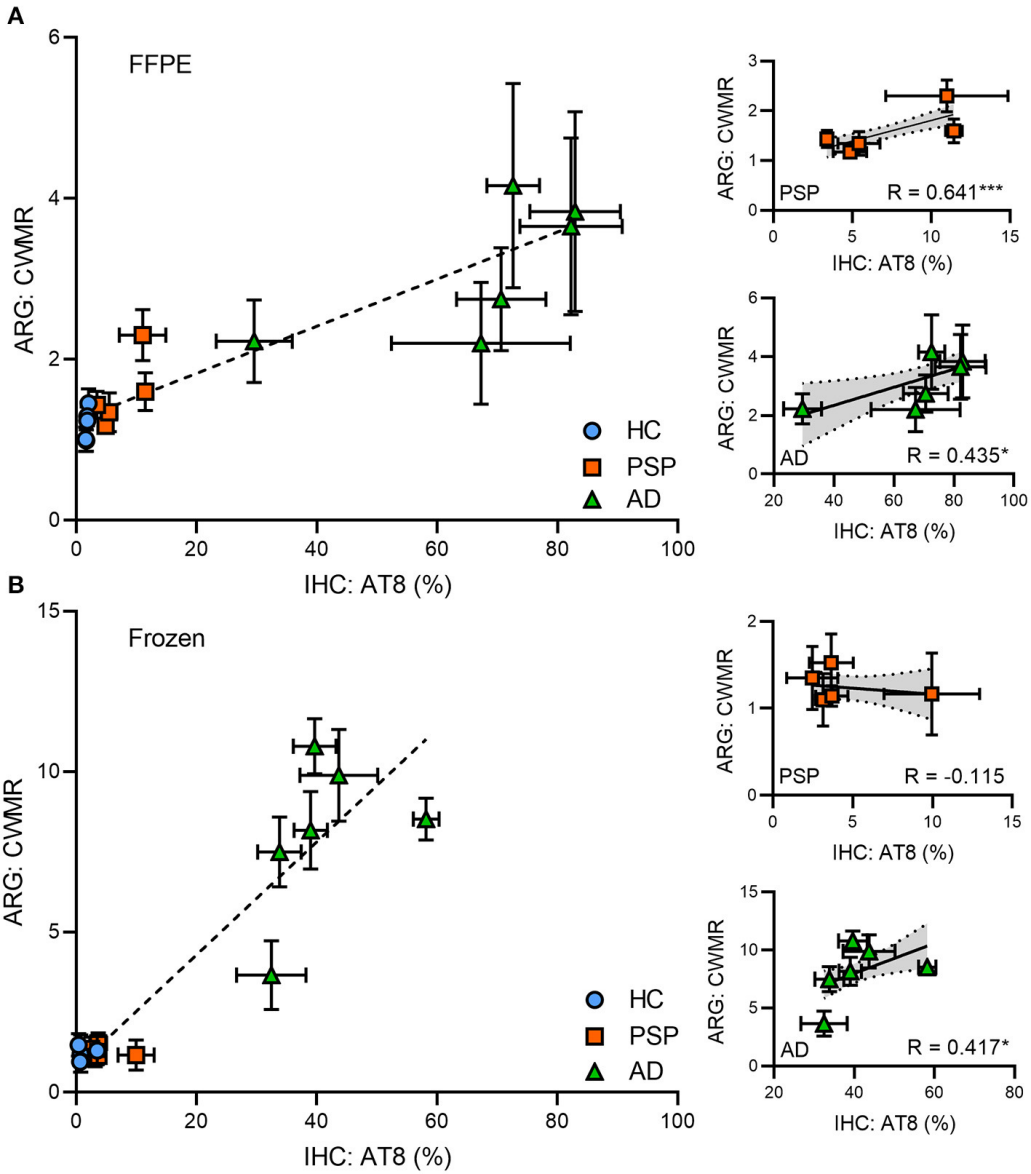
Quantitative correlation of autoradiography and immunohistochemistry for FFPE **(A)** and frozen **(B)** tissue for all entities and AD/PSP patients (each symbol represents on patient), respectively. Simple linear regressions are expressed by *R*-values and regression lines with corresponding 95%-confidence intervals for AD and PSP. AD, Alzheimer's disease; PSP, progressive supranuclear palsy; HC, healthy control; FFPE, formalin-fixed paraffin-embedded; IHC, immunohistochemistry; ARG, autoradiography; CWMR, cortex to white matter ratio; *p< 0.05, ***p < 0.001.

In FFPE samples, significant correlations between immunohistochemical tau load and relative [^18^F]PI-2620 binding in ARG were found for both PSP (*R* = 0.641, *p* < 0.001) and AD tissue (*R* = 0.435, *p* = 0.016) as illustrated in Figure 4A. In frozen tissue samples (see Figure 4B), a significant correlation was only found for AD tissue (*R* = 0.417, *p* = 0.014), whereas no significant correlation could be observed in PSP (*R* = −0.115, *p* = n.s.).

## Discussion

Several next-generation PET tracers detecting tau pathology in the human living brain have been developed (3) and were used in first clinical studies (12, 27–29). To ensure a reliable application as clinical diagnostics, it is essential to evaluate if the scan is representative of the underlying disease or suffers from off-target binding, which can lead to misinterpretation of the PET results. ARG is frequently used to investigate binding capacities of PET tracers *in vitro*, but tissue preparation prior to analysis is not standardized and might have an impact on ARG quantification. We present the first direct comparison of the next-generation tau PET tracer [^18^F]PI-2620 in both frozen and FFPE cortical brain sections from subjects with different tauopathies (AD and PSP) and HC. We show that the ARG signal correlates with the immunohistochemical tau load for both FFPE and frozen samples in AD. However, only FFPE but not frozen samples indicate a significant ARG correlation with the immunohistochemical tau load in PSP patients. This is also reflected by lacking detection of an elevated ARG binding in frozen PSP tissue when compared to healthy controls.

In our study, we were able to intra-individually compare both tissue preparation techniques, FFPE and freezing, in terms of their effects of concomitant ARG and immunohistochemistry. To our knowledge, our study represents the first direct comparison between FFPE and frozen sections with a tau radioligand. The only former study including a similar direct comparison of a β-amyloid ligand ([^18^F]florbetapir) between FFPE and frozen sections in AD samples found 43% lower binding ratios in FFPE sections when compared to corresponding frozen tissue (19). In line with this finding, binding ratios of FFPE sections were 41% lower when compared to binding ratios in frozen sections in our AD samples. Thus, lower binding ratios in FFPE sections with high target abundance seem to be independent of the tracer target (i.e., tau or β-amyloid) and may indicate higher background binding in the target-free reference tissue or less preserved binding sites of the target in FFPE sections when compared to frozen tissue.

For *in vivo* differential diagnosis of patients with suspected tauopathies, radiotracer binding to aggregated tau needs to exceed background binding of HC and other neurodegenerative diseases lacking tau pathology. Although the limited resolution of PET naturally leads to higher binding ratios of ARG sections *in vitro* when compared to relative binding of a PET tracer *in vivo* (30), ARG can still be used to predict *in vivo* PET results. In AD, [^18^F]PI-2620 indicated significantly elevated tracer binding *in vitro* (frozen sections) and *in vivo* (11, 29, 31), and ARG binding ratios of our sample proved to be significantly higher than those of HC in both FFPE and frozen sections. Taken together, both FFPE and frozen sections appear to be usable to depict specific binding to 3/4R tau isoforms in AD with significant correlations for both techniques. However, higher binding ratios in frozen tissue samples need to be considered.

It has been shown for several β-amyloid and one first-generation tau PET radiotracer that FFPE and frozen tissue preparations provide significant correlations between the immunohistochemical amyloid, respectively, tau load and the ARG signal in AD patients (11, 17–21). In line, we also found significant correlations between ARG quantification and the immunohistochemically assessed tau load of corresponding brain sections with both FFPE and frozen AD brain sections, indicating that [^18^F]PI-2620 binding has an overall high agreement with the underlying tau pathology in AD. In a recent study with several tritium labeled next-generation tau radiotracers ([^3^H]PI-2620, [^3^H]RO948, [^3^H]MK6240, and [^3^H]JNJ067), all four radiotracers depicted AD-related tau inclusions (paired helical filaments) with high specificity (11).

In the non-AD tauopathy PSP, elevated [^18^F]PI-2620 binding to PSP target regions has already been shown in a large multi-center investigation *in vivo* (12), but *in vitro* results were discrepant for FFPE (12) and frozen samples (10, 11). FFPE samples (frontal cortex, basal ganglia) from two PSP patients lacking co-pathology indicated a blockable tracer signal (12). Other studies with frozen samples only showed an elevated tracer signal when concomitant AD pathology was present (11). Our current head-to-head comparison revealed results fitting to these preliminary findings, indicating that significantly elevated ARG binding of PSP tissue (in contrast to HC) was only present in FFPE sections, whereas frozen sections did not comprise discernible binding in patients with PSP. Furthermore, a significant correlation between the [^18^F]PI-2620 ARG signal and immunohistochemical tau load was only observed in FFPE samples, whereas frozen samples did not show any association between [^18^F]PI-2620 ARG binding and AT8 quantification. While findings using tissue with both preservation types are now confirmed at least at two independent sites, the question about the origin of this discrepancy remains to be solved. First, [^18^F]PI-2620 ARG binding ratios of PSP samples were significantly lower when compared to AD samples, which can be explained by the lower tau load of deceased patients with PSP when compared to the high tau load of most of the late-stage AD cases. Furthermore, we note that different [^18^F]PI-2620 binding affinities among the underlying 4R and 3/4R tau isoforms of PSP and AD could also contribute to the lower binding ratios in PSP (31), again fitting to the observations *in vivo* (12). In this regard, the correlation between AT8 staining and [^18^F]PI-2620 ARG binding ratios of FFPE tissue gave a satisfactory fit when considering PSP and AD samples together (Figure 4A). Thus, the resulting signal per amount of tau seems at least roughly comparable between 4R and 3R/4R tauopathies. Importantly, micro-ARG of PSP tissue confirmed that [^3^H]PI-2620 binding was co-localized with tau (31), which makes a FFPE induced off-target source unlikely. Furthermore, the significant correlation between binding in FFPE samples of PSP patients and immunohistochemically assessed tau load supports the claim that the ARG signal is specific, but larger sample sizes are needed to confirm those assumptions.

Still, the lacking [^18^F]PI-2620 ARG signal of frozen PSP sections deserves further discussion. Since tissue preparation followed the same standardized protocol for all samples (HC, PSP, AD) and AD patients showed even higher binding in frozen samples, we conclude that the discrepant results cannot be explained by the preparation techniques themselves. We speculate that the higher density of tau in AD neurofibrillary tangles could contribute to a better preservation of binding sites in frozen tissue when compared to PSP. In PSP, the lower amount and the more diffuse type of tau, which is not only located in neuronal bodies but also in faint processes of astroglia might be less preserved in frozen tissue. Although we avoided to compare AT8 quantification between FFPE and frozen sections due to potential interhemispheric differences, our data would at least roughly support this explanation since AT8 quantification was consistently lower in frozen PSP tissue (mean load 4.6%) when compared to FFPE PSP tissue (mean load 7.2%). In conclusion, the fixation related preservation of faint tau aggregation may lead to detectable ARG binding only in FFPE but not in frozen tissue.

### Limitations

To avoid bias by co-pathology, we searched for samples without concomitant α-synuclein, TDP-43 or FUS in our brain bank. Thus, the resulting sample sizes of our study were limited, consisting of five HC, five PSP and six AD patients. Yet, all samples comprised both frozen and FFPE brain sections, which represents a strength of this head-to-head comparison. To enhance reproducibility and to increase the robustness of our data, multiple samples for both tissue techniques were used from each patient and five cortical regions-of-interest from each section were included in the analysis. PSP patients indicated significantly higher cortex to white matter binding ratios compared to HC, but lower cortex to white matter binding ratios when compared to AD. In contrast to AD, tau pathology of PSP is not limited to neurons but is also present in glial cells and can therefore also be present in white matter regions. Although we selected reference tissue devoid of a positive AT8 signal, we cannot fully exclude a remaining impact of subcortical tau pathology on our quantification.

Not all target regions known to be affected in AD/PSP are reflected in this study. As unspecific binding, e.g., to MAO-B, has been shown for first-generation tau radiotracers especially in subcortical brain regions (5), frontal cortex samples were used for this head-to-head comparison. Further studies should also consider cortical and subcortical brain regions in order to expand the current results.

As the amount of tau pathology can vary between different hemispheres and FFPE/frozen samples are taken from one hemisphere each of the same donor, a direct quantitative comparison of immunohistochemistry and autoradiography results between both is hampered.

Another limitation is the concentration of tracer used. In the ARG experiments described here, a concentration of 1.6 nM of [^18^F]PI-2620 was used for comparability between AD and PSP samples. This concentration is in the range of the IC50 for AD, but seems to be lower than the IC50 described for PSP (31). Especially in frozen samples with potential structural loss as hypothesized above, the lower concentration could contribute to the reduced binding in PSP samples. Higher concentrations should be tested in subsequent experiments and analyses.

## Conclusion

In postmortem tissue of AD patients, FFPE and frozen brain samples can be used for *in vitro* evaluation of the novel next-generation tau-radiotracer [^18^F]PI-2620. Frozen samples of PSP patients did not indicate specific cortical binding of [^18^F]PI-2620, whereas the ARG signal of FFPE samples significantly correlated with the immunohistochemical tau load. Therefore, FFPE samples should be favored for further investigation of binding capacities of [^18^F]PI-2620 in non-AD tauopathies by ARG.

## Data Availability Statement

The raw data supporting the conclusions of this article will be made available by the authors, without undue reservation.

## Ethics Statement

The studies involving human participants were reviewed and approved by LMU Munich application number 19-244. Written informed consent for participation was not required for this study in accordance with the national legislation and the institutional requirements.

## Author Contributions

MW, SR, MB, and LB work was designed. MW, SR, AH, TA, MS, GR, and GH contributed to acquisition of data and/or analysis. MW, SR, AM, NK, MB, and LB data and results were interpreted. MW, SR and LB drafted the work. GR, OS, HB, MP, OM, AM, NK, ASc, ASt, GH, PB, JH, MB and LB substantively revised the work. All authors read and approved the final manuscript.

## Conflict of Interest

GH has served on the advisory boards of UCB and Biogen. JL reports speaker fees from Bayer Vital and Roche, consulting fees from Axon Neuroscience and Ionis Pharamceuticals, author fees from Thieme medical publishers and W. Kohlhammer GmbH medical publishers, non-financial support from Abbvie and compensation for duty as part-time CMO from MODAG, outside the submitted work. OS received research support from Life Molecular Imaging. MB received speaker honoraria from GE healthcare and LMI and is an advisor of LMI. AM, NK, and ASt are employed by Life Molecular Imaging. The remaining authors declare that the research was conducted in the absence of any commercial or financial relationships that could be construed as a potential conflict of interest.
