# Supplementary material for: Superiority of Formalin-Fixed Paraffin-Embedded Brain Tissue for in vitro Assessment of Progressive Supranuclear Palsy Tau Pathology With [18F]PI-2620
Source: Front Neurol. 2021 Jul 2;12:684523. doi: 10.3389/fneur.2021.684523 (PMC8282895; doi:10.3389/fneur.2021.684523)
Supplement: Supplementary file 1 [file Data_Sheet_1.PDF]

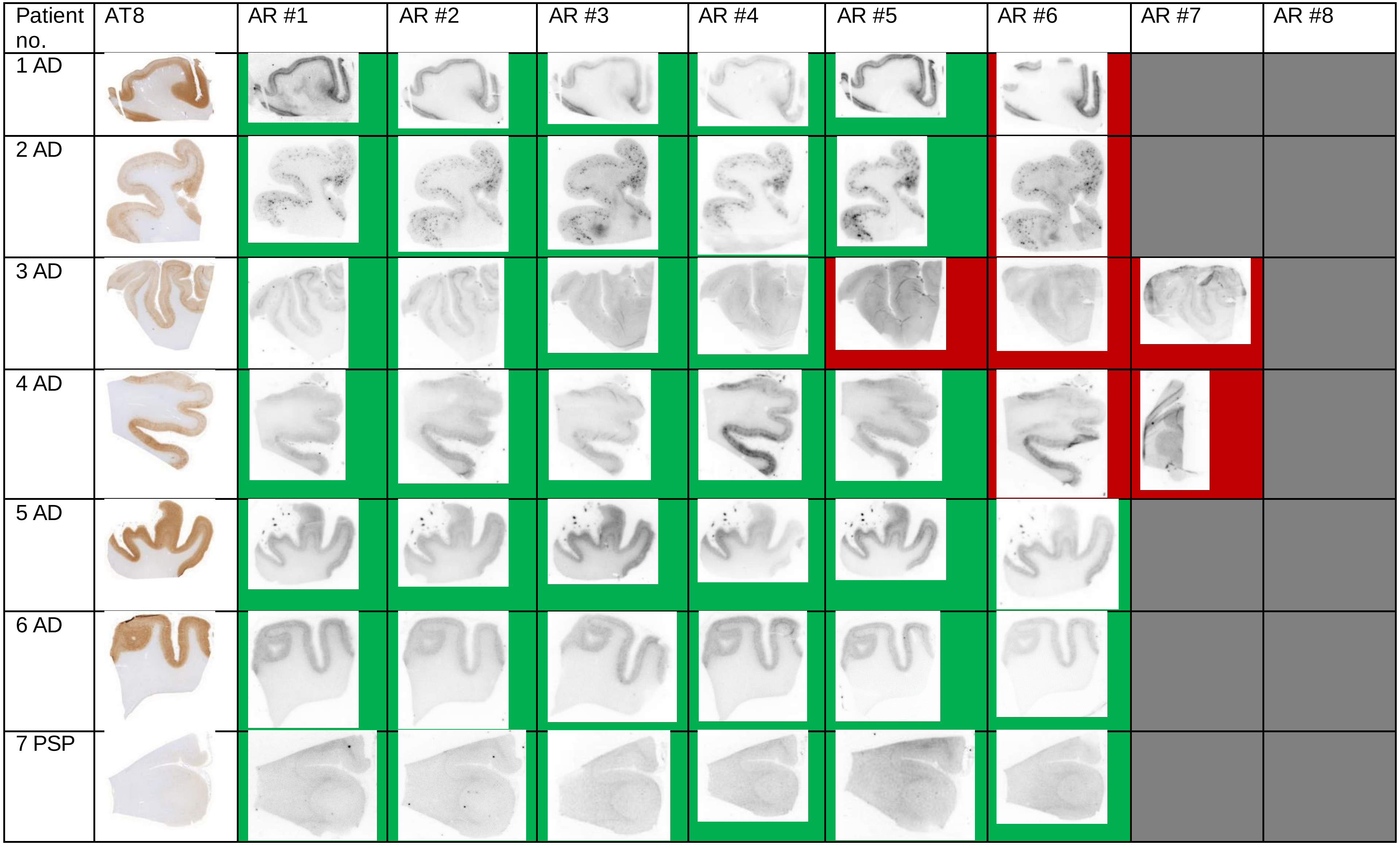
Patient no.
AT8
AR #1
AR #2
AR #3
AR #4
AR #5
AR #6
AR #7
AR #8
1 AD
2 AD
3 AD
4 AD
5 AD
6 AD
7 PSP

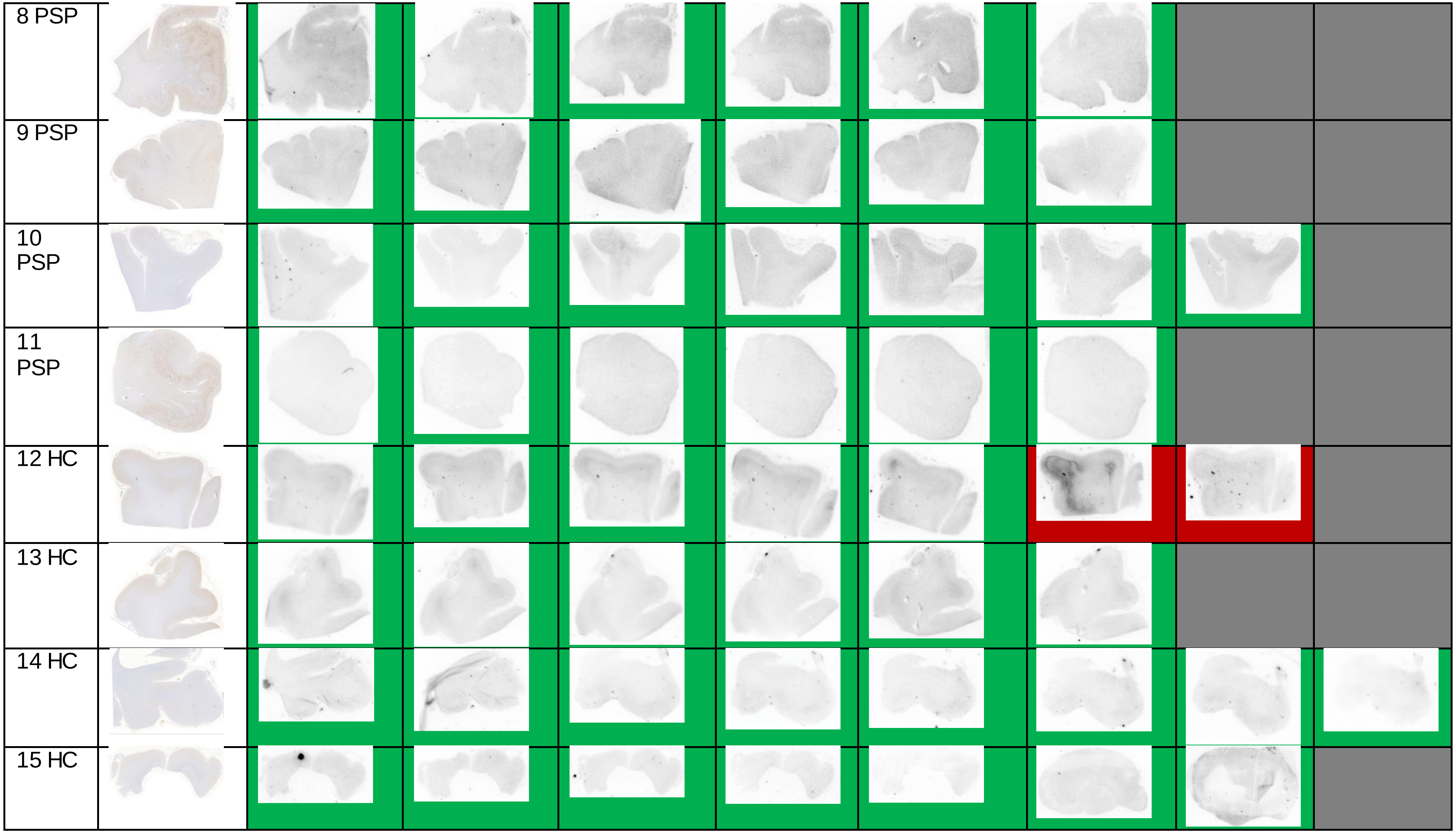
8 PSP
9 PSP
10 PSP
11 PSP
12 HC
13 HC
14 HC
15 HC

16 HC		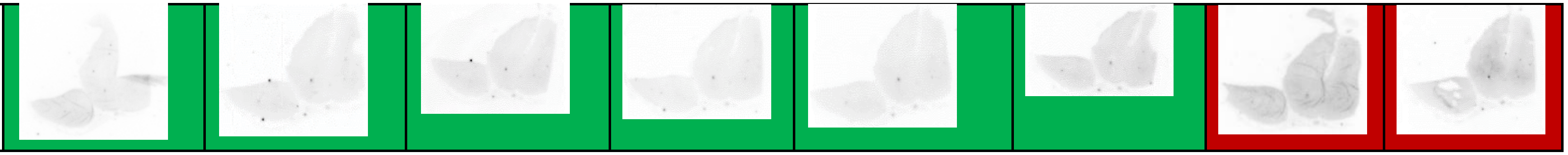							

Supplemental Table 1: Formalin-fixed paraffin-embedded brain sections. All brain sections included in the AR analysis are highlighted in green, all excluded brain sections are highlighted in red. AR, autoradiography. AD, Alzheimer's disease. PSP, progressive supranuclear palsy. HC, healthy control.

Patient no.	AT8	AR #1	AR #2	AR #3	AR #4	AR #5	AR #6	AR #7	AR #8
1 AD	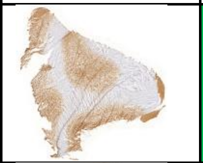	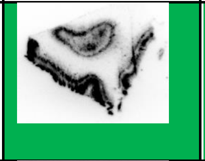	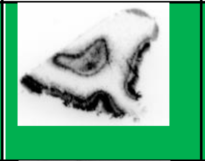	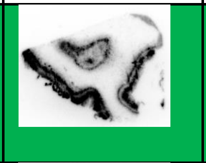	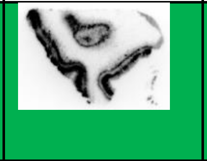	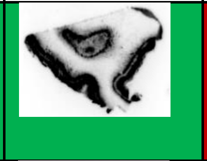	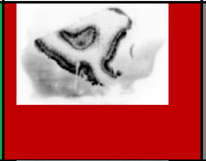		
2 AD	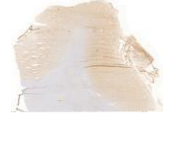	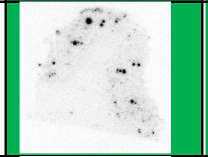	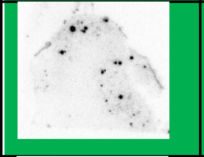	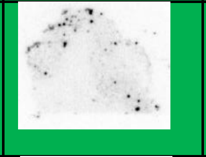	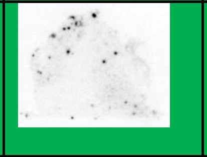	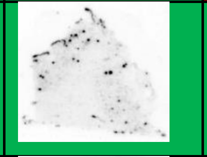	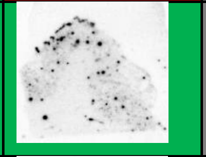		
3 AD	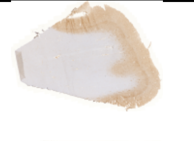	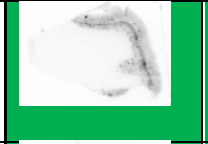	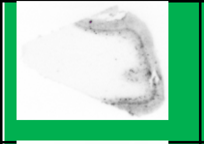	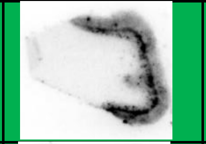	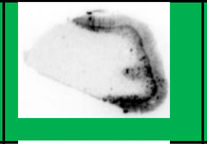	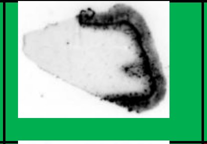	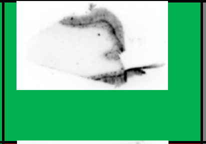		
4 AD	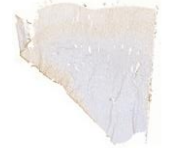	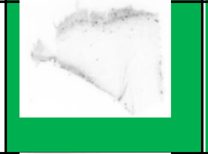	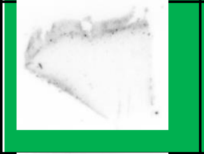	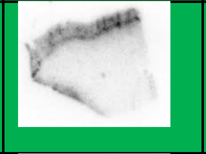	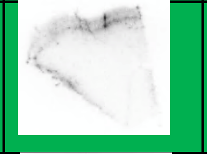	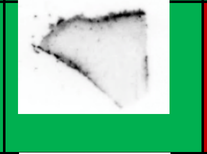	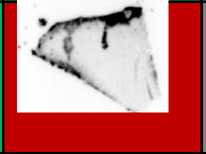		
5 AD	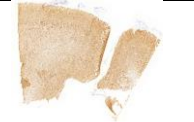	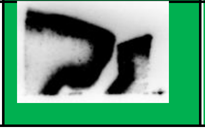	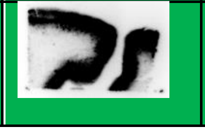	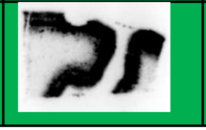	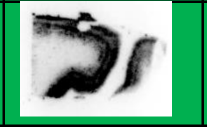	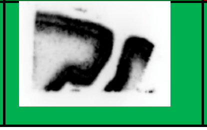	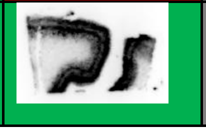		

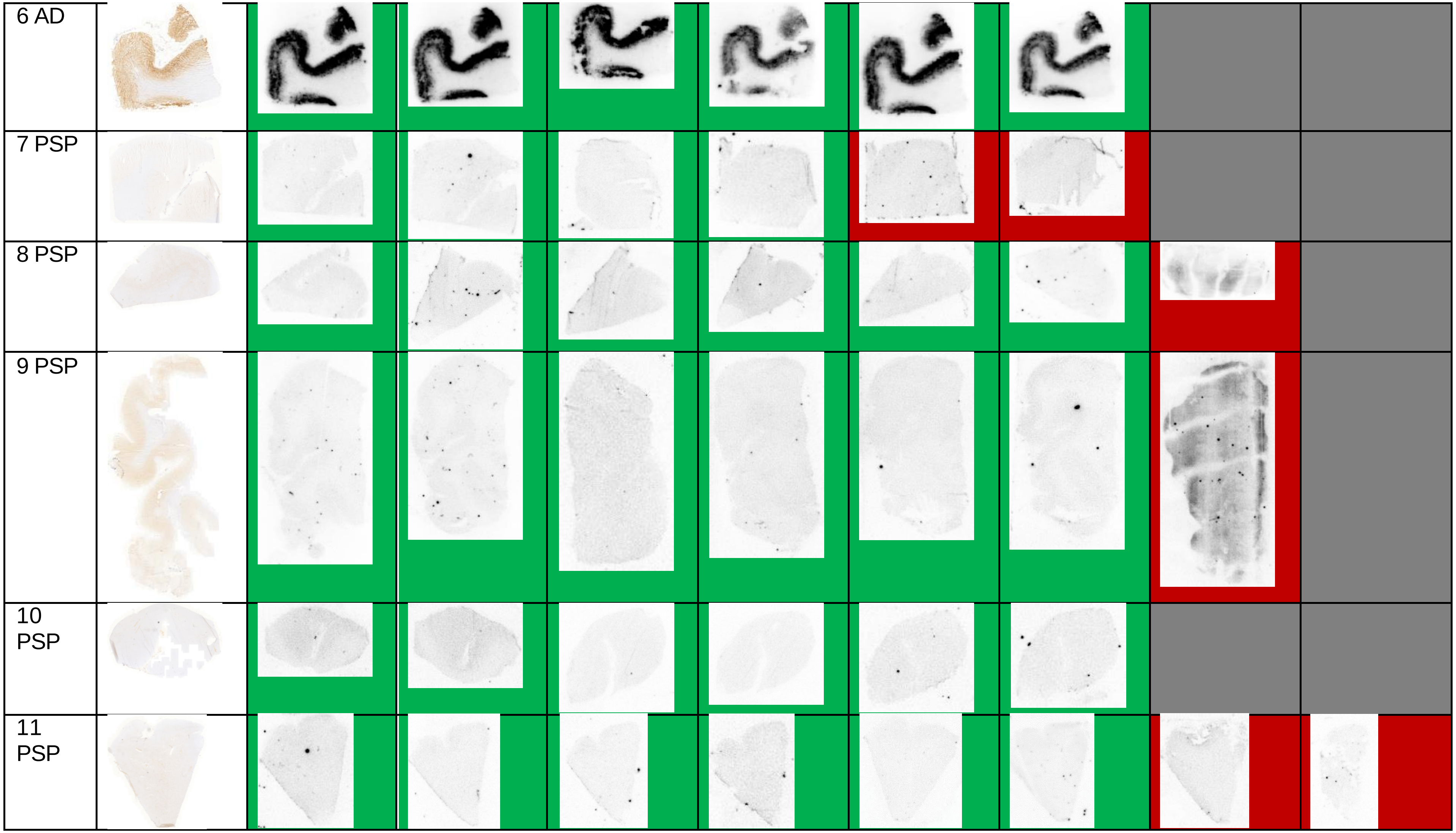
6 AD
7 PSP
8 PSP
9 PSP
10 PSP
11 PSP

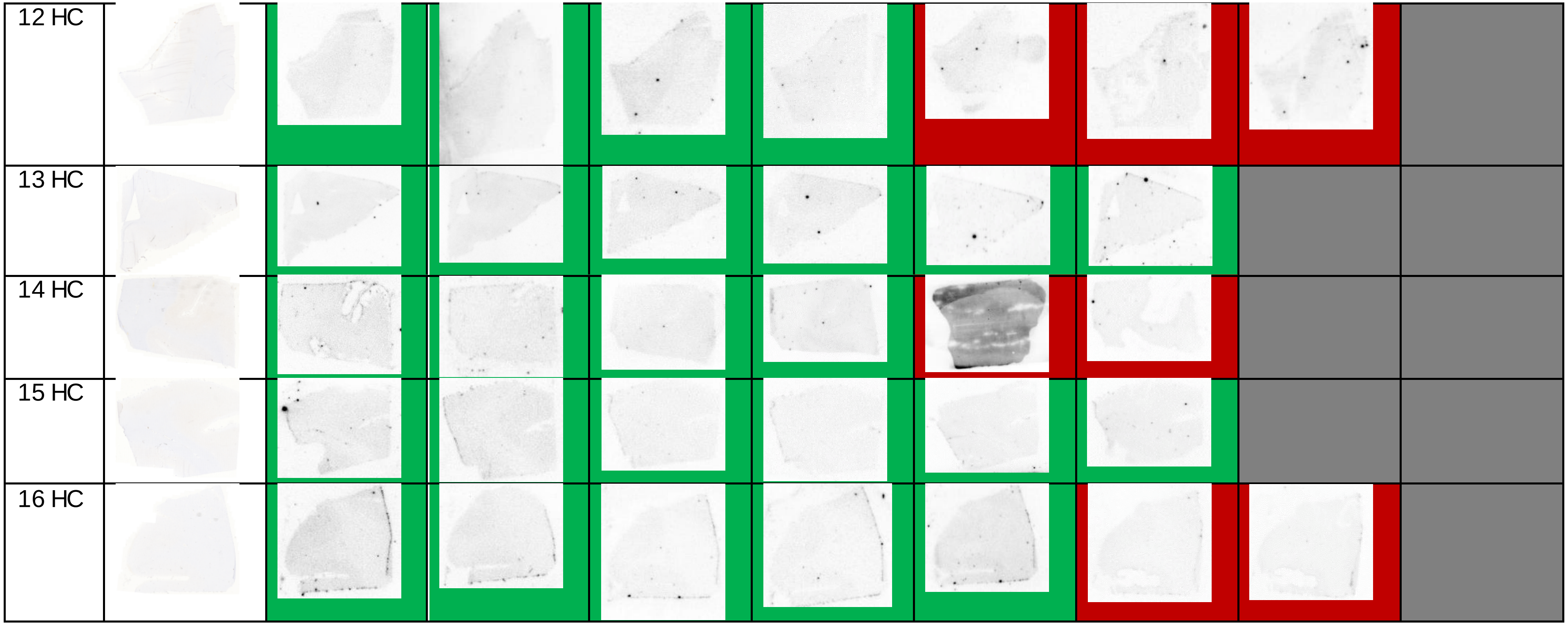

Supplemental Table 2: Frozen brain sections. All brain sections included in the AR analysis are highlighted in green, all excluded brain sections are highlighted in red. AR, autoradiography. AD, Alzheimer‘s disease. PSP, progressive supranuclear palsy. HC, healthy control.
